# Supplementary material for: The dynamic transmission of positional information in stau- mutants during Drosophila embryogenesis
Source: eLife. 2020 Jun 8;9:e54276. doi: 10.7554/eLife.54276 (PMC7332292; doi:10.7554/eLife.54276)
Supplement: Figure 1—source data 2. — Source data for Figure 1E. [file elife-54276-fig1-data2.docx]

Figure 1-source data 2. The dynamics of the average and variability of Hb boundaries with equal sample size binning in the WT and *stau^-^* mutants on both the dorsal and ventral sides**.** Total sample numbers for WT and *stau^-^* mutants are 47 and 69, respectively. The sample size of each bin is 7 and 10 for the WT and *stau^-^* mutants, respectively except the last one. Errors represent the stand deviation of each bin and their uncertainty in the parentheses is calculated with bootstrap.

| t(min) | x_hb_ (WT) (%EL) | | x_hb_ (*stau^-^*) (%EL) | | |
| --- | --- | --- | --- | --- | --- |
|  | Dorsal | Ventral | | Dorsal | Ventral |
| 5 | 45.4±1.7 (4) | 43.1±1.0 (2) | | 38.2±2.1 (2) | 33.1±1.9 (4) |
| 14 | 46.6±1.7 (4) | 43.4±1.7 (3) | | 41.2±2.1 (4) | 34.4±2.1 (3) |
| 25 | 47.5±1.2 (3) | 44.3±2.4 (8) | | 43.4±1.2 (3) | 37.9±1.2 (4) |
| 34 | 47.6±1.4 (2) | 44.6±0.6 (2) | | 45.2±1.3 (3) | 40.1±1.3 (5) |
| 43 | 47.2±1.2 (3) | 43.9±1.0 (2) | | 46.8±1.3 (2) | 40.7±1.3 (3) |
| 51 | 47.8±0.9 (2) | 43.6±0.8 (2) | | 47.1±1.5 (3) | 41.4±1.5 (5) |
| 57 | 47.2±1.4 (7) | 43.9±2.3 (7) | | 48.1±0.9 (2) | 43.9±0.9 (3) |
