# Supplementary material for: The dynamic transmission of positional information in stau- mutants during Drosophila embryogenesis
Source: eLife. 2020 Jun 8;9:e54276. doi: 10.7554/eLife.54276 (PMC7332292; doi:10.7554/eLife.54276)
Supplement: Figure 5—source data 1. — Source data for Figure 5. [file elife-54276-fig5-data1.docx]

Figure 5- source data 1. Comparison of the minimal positional errors of patterning markers including average Bcd gradients at 16 min into nc14 after subtracting the imaging noise and image mask noise, posterior boundary of the anterior Hb domain at 46 min (WT) or 51 min (*stau^-^* ) into nc14, the anterior boundary of Kr at 40 min into nc14, the 1^st^ peak of Eve at 47 min (WT) or 38 min (*stau^-^* ) into nc14, and CF at 56 min into nc14. The standard deviation of the average positional noise is calculated based on bootstrapping.

| **Patterning markers**    **Genotypes** | **Bcd** | **Hb** | **Kr** | **Eve** | **CF** |
| --- | --- | --- | --- | --- | --- |
| WT (%EL) | 2.6±0.4 | 0.9±0.3 | 0.7±0.1 | 0.9±0.1 | 1.4±0.1 |
| *stau^-^* (%EL) | 3.0±0.5 | 1.1±0.5 | 1.2±0.1 | 1.0±0.1 | 1.1±0.1 |
