# Supplementary material for: The dynamic transmission of positional information in stau- mutants during Drosophila embryogenesis
Source: eLife. 2020 Jun 8;9:e54276. doi: 10.7554/eLife.54276 (PMC7332292; doi:10.7554/eLife.54276)
Supplement: Figure 6—source data 1. [file elife-54276-fig6-data1.docx]

| Parameter | | WT | Bcd1.0 | *stau-* | Source |
| --- | --- | --- | --- | --- | --- |
| Hb degradation rate | $\beta$ | $0.1 {min}^{-1}$ | | | (Papatsenko and Levine, 2008) |
| Hb diffusion rate | $D$ | $12 {\mu m}^{2}/min$ | | | (Papatsenko and Levine, 2008) |
| Bcd decay rate | $\omega_{0}$ | $0.01 {min}^{-1}$ | | | (Little et al., 2011) |
| Hill coefficient for Bcd | $n_{b}$ | $5$ | | | (Driever et al., 1989; Gregor et al., 2007a) |
| Hill coefficient for Hb | $n_{h}$ | $3$ | | | (Treisman and Desplan, 1989) |
| Bcd gradient amplitude | $b_{m}$ | 2 | $1$ | $0.70$ | Measured |
| Bcd length constant | $\lambda$ | $0.15 EL$ | | $0.18 EL$ |  |
| Amplitude of initial Hb | $h_{m}$ | 1 | | | Fixed |
| Steepness of initial Hb boundary | $k_{x}$ | $16{EL}^{-1}$ | | |  |
| Bcd regulation strength | $\alpha_{b}$ | $0.23 {min}^{-1}$ | | | [0.1~0.5] |
| “Threshold” for Bcd | $b_{0}$ | $0.10$ | | | [0.01~0.31] |
| Hb regulation strength | $\alpha_{h}$ | $0.27 {min}^{-1}$ | | | [0.1~0.5] |
| “Threshold” for Hb | $h_{0}$ | $1.20$ | | | [1~2] |
| Time triggering Bcd decay | $t_{0}$ | $4.59 min$ | | | [-5~10] |
| Simulation time offset of the beginning of nc14 | $t_{offset}$ | $-10 min$ | | | [-20~20] |
| Boundary position of initial Hb distribution | $x_{0}$ | $0.30 EL$ | | $\to\infty$  (Fixed) | [0.3~0.5] |
